# Salvage therapy of pretreated advanced breast cancer with bevacizumab and paclitaxel every two weeks: a retrospective case review study

**DOI:** 10.1186/1471-2407-9-338

**Published:** 2009-09-23

**Authors:** Alexandros Ardavanis, Dimitrios Doufexis, Panteleimon Kountourakis, Savvoula Malliou, Athanasios Karagiannis, Evgenia Kardara, Despina Sykoutri, Margari Charalampia, Gerasimos Rigatos

**Affiliations:** 1 Department of Medical Oncology, St Savas Anticancer hospital, Athens, Greece

## Abstract

**Background:**

Targeting angiogenesis is nowadays one of the most promising approaches for breast cancer. Bevacizumab (BEV), a VEGF-trap monoclonal antibody, was recently approved in combination with paclitaxel (PAC) for the first line treatment of advanced breast cancer (ABC). The activity of this combination in pretreated patients is not known.

**Methods:**

Patients with pretreated ABC and progressive disease received BEV 10 mg/kg with PAC 135 mg/m^2 ^every two weeks for six months and then maintenance with BEV 15 mg/kg every three weeks until progression. This regimen was chosen for better patient convenience, while maintaining the same dose intensity for both drugs.

**Results:**

42 patients were reviewed retrospectively (41 f, 1 m, mean age 57 years). Overall response rate was 35.7%. Stable disease was observed in 45.2% of patients, whereas 14.3% of patients progressed. The median overall survival was greater than 20 months, with a one year rate of 83.4%. The median progression free survival was 12.1 months, with a one year rate of 51.8%. Toxicity was in general acceptable.

**Conclusion:**

This biweekly BEV/PAC combination seems to be active with acceptable toxicity in pretreated ABC with an advantage over the weekly regimen regarding quality of life and preservation of resources.

## Background

Cancer is a major public health problem in most parts of the world. It is estimated that about 565,650 Americans died from cancer in 2008 and among these deaths, 40,480 were due to breast cancer in women. The new breast cancer cases among American women for 2008 were 182,460 [1].

Despite adjuvant chemotherapy, approximately 40--50% of patients will develop recurrent and/or metastatic breast cancer with angiogenesis, playing a central role in both local tumour growth and distant metastasis formation [2]. Multiple angiogenic factors are commonly expressed by invasive breast cancers and the 121-amino-acid isoform of vascular endothelial growth factor (VEGF) predominates [3].

Bevacizumab (BEV) (Avastin^©^, F. Hoffmann-La Roche Ltd) is a recombinant humanized monoclonal antibody against VEGF. Preclinical in vivo models demonstrate that BEV inhibits growth of a variety of human cancer cell lines in a dose dependent manner. In addition, by eliminating the excess VEGF, the newly formed tumour vessels become less permeable, resulting thus in reduction of interstitial pressure. It has been shown that the latter effect increases the diffusion of chemotherapeutic drugs in the tumour and perhaps potentiates their activity [4].

In general, combination chemotherapy results in higher response rates, longer times to progression [5] and in some studies prolongs overall survival [6], when compared to single agent therapy. In a phase III trial, the addition of BEV to capecitabine in patients previously treated with anthracyclines and taxanes significantly increased the objective response rate (9.1% vs. 19.8%, *p *= 0.001) but not progression-free survival (4.2 vs. 4.9 months; hazard ratio for disease progression, 0.98) or overall survival (15.1 vs. 14.5 months) [7]. In another phase III trial by Miller et al., paclitaxel plus bevacizumab significantly prolonged progression-free survival as compared with paclitaxel alone (median, 11.8 vs. 5.9 months; *p *< 0.001) and increased the objective response rate (36.9% vs. 21.2%, *p *< 0.001) in the first line setting. The overall survival rate, however, was similar in the two groups (median, 26.7 vs. 25.2 months; *p *= 0.16) [8].

The combination of BEV and PAC seems to be a promising combination and has been recently approved as initial therapy for patients with ABC [8], nonetheless, literature data regarding this combination in the 2^nd^+ line of treatment are scarce.

In an effort to estimate the potential benefit of a prospective, randomized controlled trial comparing the combination of BEV and PAC to PAC alone in pre-treated patients, we analysed the data of patients with ABC in the 2^nd^+ line of treatment, who received a modified biweekly regimen of BEV and PAC.

## Methods

### Patient eligibility and baseline evaluation

The population included male and female patients, 18 years of age or older, with histologically or cytologically confirmed ABC, who had received at least one line of cytotoxic treatment for metastatic disease. Minimum time interval from last administration of taxanes required for inclusion was 9 months. For this retrospective analysis of patient records, approval was obtained from the Institutional Review Board (IRB,7996/361).

Additional inclusion criteria included Eastern Cooperative Oncology Group (ECOG) performance status of 0 or 1, no significant cardiovascular disease, no previous thromboembolic disease and adequate renal, hepatic, and hematologic function. The presence of measurable tumour was not required for inclusion. Patients were excluded if they had had another cancer within a period of 5 years before initiation of 2^nd ^line treatment, major surgery within a period of 4 weeks before initiation of 2^nd ^line treatment, or if they had a non-healing wound or fracture, an infection requiring parenteral antibiotics at the time of initiation of 2^nd ^line treatment. Patients were excluded if they were on therapeutic anticoagulant agents, non-steroidal anti-inflammatory agents. Use of prophylactic low-dose anticoagulant agents wasn't considered as an exclusion criterion.

Pre-treatment evaluation included a complete medical history and physical examination, a full blood count, a biochemical profile (SGOT, total bilirubin, creatinine), prothrombin time, INR, and partial thromboplastin time. In addition, computed tomography (CT) of the chest, abdomen and brain or magnetic resonance imaging (MRI), and assessment of performance status according to ECOG were performed [9]. A complete blood count was obtained regularly. An interim medical history, physical examination and the laboratory tests listed above were repeated prior to the start of each cycle of therapy.

### Treatment plan

All patients received BEV 10 mg/kg with PAC 135 mg/m^2 ^with appropriate premedication and supportive care, every two weeks for six months and then maintenance with BEV 15 mg/kg every three weeks until progression. This regimen was chosen instead of the established one (weekly PAC) for better patient convenience, while maintaining the same dose intensity of the established weekly regimen for both drugs. Treatment was interrupted in case of proteinuria (urinary protein excretion, ≥ 2000 mg per 24 hours). Antihypertensive therapy was administered at the discretion of the treating physician. BEV therapy was not withheld or discontinued for PAC-related toxic effects. The patients continued therapy until disease progression or prohibitive toxic effects occurred. Patients who discontinued PAC without disease progression (i.e., because of toxic effects or at the discretion of the patient or investigator) continued BEV monotherapy until disease progression or unacceptable toxic effects occurred.

### Safety and efficacy

Toxicities were evaluated on days 1 and 15 of each treatment cycle and graded according to the National Cancer Institute Common Toxicity Criteria NCI-CTC, version 2.0. Tumour response was determined by the treating physicians according to WHO criteria [10] at baseline and every 12 weeks until disease progression.

### Statistical analysis

Our analysis objective of was the preliminary assessment of the efficacy and toxicity profile of the combination of BEV and PAC in patients with ABC.

Data are expressed as median and min-max for continuous variables and as percentages for categorical data. The Kolmogorov---Smirnov test is utilized for normality analysis of the parameters. Overall survival (OS) and progression-free survival (PFS) are estimated with the Kaplan-Meier product--limit method [11]. All tests are two-sided with 95% significance level. The statistical analysis was carried out using the statistical package SPSS ver. 15.00 (Statistical Package for the Social Sciences, SPSS Inc., Chicago, Ill., USA).

## Results

### Patients

41 female and 1 male patients, who presented with ABC between January 2007 and December 2008 were reviewed. Median age was 57.5 years (range 33-82 years). All patients had previously received cytotoxic chemotherapy with taxanes and/or anthracyclines for metastatic disease. HER-2 positivity was noticed in 15 patients (35.7%). The median number of cycles of paclitaxel administered was 9 (range 3-12) and of bevacizumab was 21 (3-29). A summary of baseline patient characteristics is shown in Table 1. No patient was withdrawn from the study.

**Table 1:** Baseline patient and disease characteristics

	No. of Patients (n = 42)	%
Median Age (years)	57.5	
Range	33-82	
HER2 status		
Negative	27	64.3
Positive	15	35.7
Metastatic disease		
Bone	21/42	50
Liver	17/42	40.5
Skin	16/42	38.1
Lung	12/42	28.6
CNS^†^	5/42	11.9
Lymph nodes	4/42	9.5
Bone marrow	1/42	2.4
Malignant pleural effusion	4/42	9.5
Malignant ascites	1/42	2.4
Line of treatment		
2^nd^	21	50
3^rd^	15	35.7
4^th^	4	9.5
5^th^	2	4.8

^†^CNS: Central nervous system

### Toxicity

Grade I neutropenia was encountered in 5 patients (11.9%) and grade II or III in 7 patients (16.6%). Only one patient (2.4%) developed grade IV neutropenia and one was diagnosed with febrile neutropenia. In 4 patients with grade III or IV neutropenia the treatment dose was reduced by 10%. Low grade anaemia (I-II) was observed in 4 patients (9.5%) and grade I-III neuropathy was seen in 33.3% of patients, with grade II neuropathy being the most frequently occurring (6 patients, 14.3%). Hypertension was seen in 16.7% of patients and was managed medically. Proteinuria was rarely clinically significant. Thromboembolic events were infrequent overall; however, there was one death recorded due to cerebrovascular ischaemia (stroke) related to treatment. The treatment related adverse events are summarised in Tables 2 &3.

**Table 2:** Treatment related adverse events

	No. of Patients (n = 42)			
	
	Grade I	Grade II	Grade III	Grade IV
Haematological				
Anaemia	3 (7.1%)	1 (2.4%)	-	-
Neutropenia	5 (11.9%)	4 (9.5%)	3 (7.1%)	1 (2.4%)
Neuropathy	5 (11.9%)	6 (14.3)	3 (7.1%)	-

**Table 3:** Treatment related adverse events

	No. of patients (n = 42)	%
Hypertension (Grade I)	7	16.7
Proteinuria (Grade II)	2	4.8
Febrile neutropenia (Grade III)	1	2.4
Arterial thrombosis (Grade II)	1	2.4
Venous thrombosis (Grade II)	1	2.4
Toxicity related deaths	1	2.4

### Efficacy

**Figure 1:**
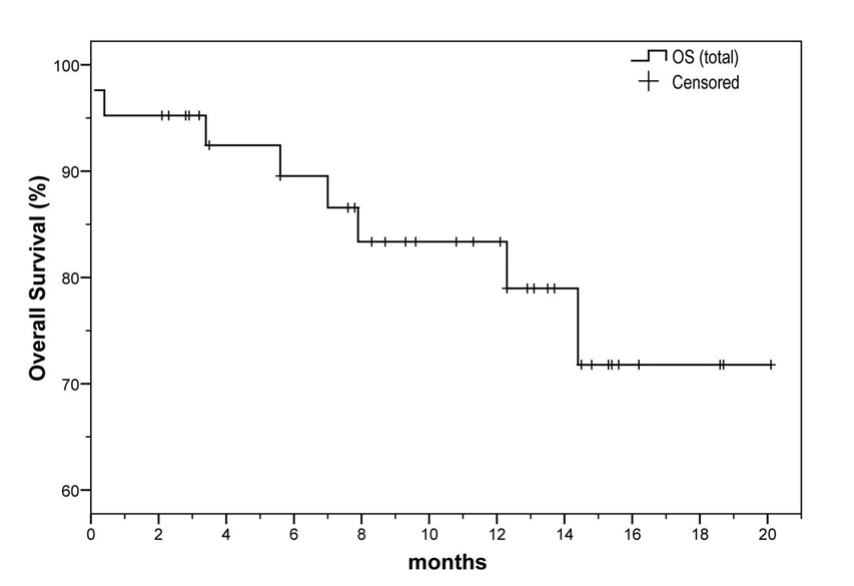
**Total overall survival (OS) of patients with ABC in the 2^nd^+ line of treatment, who received a modified biweekly regimen of BEV and PAC**.

**Figure 2:**
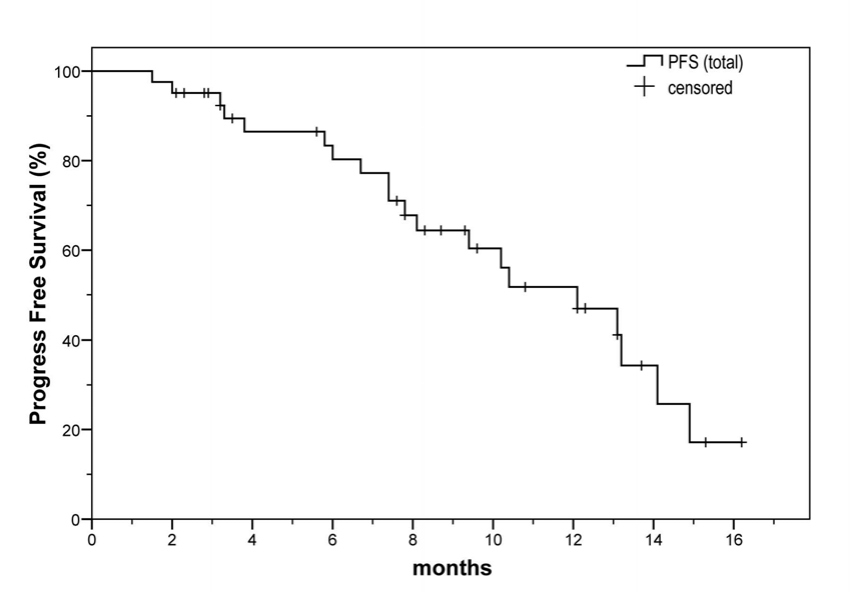
**Total progression free survival (PFS) of patients with ABC in the 2^nd^+ line of treatment, who received a modified biweekly regimen of BEV and PAC**.

Out of the 42 patients, 3 patients (7.1%) achieved complete response, while 12 patients (28.6%) had partial response, leading to an overall response rate of 35.7%. Impressive responses were noticed in skin lesions and two remissions in CNS lesions were recorded. Finally, stable disease was observed in 19 patients (45.2%), whereas 6 patients (14.3%) progressed (Table 4). At 20 months of follow-up, the projected (Kaplan-Meier) overall survival was approximately 70%. This implies that the median OS is greater than 20 months, with a one year OS rate of 83.4% (Figure 1, Table 5). The median progression free survival (PFS) was 12.1 months, with a one year PFS rate of 51.8% (Figure 2, Table 5).

**Table 4:** Response rates

	Patients (n = 42)
Response (WHO criteria)	
Complete	3 (7.1%)
Partial	12 (28.6%)
**Overall**	
**(Complete + Partial)**	**15 (35.7%)**
Stable Disease	19 (45.2%)
Progressive Disease	6 (14.3%)
Missing data	2 (4.8%)

**Table 5:** Overall and progression free survival.

	Total
OS	
Median	> 20 months
6 month	89.5%
12 month	83.4%
PFS	
Median (months, 95% CI)	12.1 (8.4-15.8)
6 month	80.3%
12 month	51.8%

## Discussion

In our analysis, 42 patients with metastatic breast carcinoma previously treated with taxanes and/or anthracyclines based regimens and who received a biweekly, combination regimen of BEV and PAC as 2^nd^+ line of treatment, were reviewed. Overall response rate was 35.7% and disease progression rate was 14.3%. The median OS was greater than 20 months and the median PFS was 12.1 months. The treatment was generally well tolerated and toxicity was acceptable. Only one death was recorded due to toxicity.

Anthracyclines and taxanes are the most effective agents for treatment of hormone receptor-negative breast cancer. The taxanes, PAC and docetaxel, have been found to be as efficacious as anthracyclines in the first-line therapy for ABC [5,12,13]. Even in second- and third-line settings, PAC has demonstrated single-agent response rates of 35% to 53% and response durations ranging from 6.8 to 7.5 months [14,15]. However, the widespread use of anthracyclines and taxanes, especially PAC, in the adjuvant setting has led to an increasing number of patients presenting with advanced disease that is resistant or intolerant to both drugs. Although there is no standard chemotherapy for patients with ABC after failure of anthracyclines and taxanes, combination chemotherapy is the most commonly used method of treatment [16].

In a phase I/II study of 75 pretreated patients with ABC, BEV revealed clinical activity as a single agent, with an objective response rate of 9.3%; 17% of patients had a response or were stable at 22 weeks [17]. A phase III trial of capecitabine (2500 mg/m^2^/d on day 1--14 every 3 weeks) alone or in combination with BEV (15 mg/kg on day 1) in 462 patients previously treated with both an anthracycline and a taxane was well tolerated and significantly increased the response rate (19.8% *vs. *9.1%; *p *= 0.001). Nevertheless, this did not result into improved progression free survival (PFS) (4.86 *vs. *4.17 months) or OS (15.1 *vs. *14.5 months). It was hypothesised that the optimal time to use an anti-angiogenic agent might be earlier in the course of disease [7]. In the pivotal E2100 phase III trial 722 previously untreated patients with ABC were randomised to PAC 90 mg/m^2 ^on days 1, 8, 15 of a 28-day cycle with or without BEV 10 mg/kg on days 1 and 15. The tolerability of the combination therapy was acceptable; hypertension rate requiring therapy on the BEV arm was 15% *vs. *1.4% in the PAC group. PFS (11.4 *vs. *6.1 months, *p *< 0.0001) and overall response rate (29.8% *vs. *13.8%, *p *< 0.0001) were significantly increased in the combination group. Notably, there was a trend towards improved OS in the combination arm (26.7 vs. 25.2 months; *p *= 0.16); nevertheless, longer follow-up is needed to demonstrate a benefit in survival [8]. Currently, there are insufficient data in the literature regarding the use of the PAC and BEV combination in pretreated patients with ABC. Our results show that the combination of BEV and PAC in this patient population leads to an overall response rate of 35.7%, median OS greater than 20 months and median PFS of 12.1 months. The presence of a significant percentage of metastases with more favourable prognosis among our patients (skin, bone, lungs), might in part explain the better, as compared to previous reports, results observed; moreover, the lower than reported in the literature percentage of HER2-negative patients (eventually including dismal phenotypes, as Basal-like, Normal-like) may also be responsible for the somewhat improved results, although interpretation of this observation is equivocal based on current knowledge. Finally, response of evaluable-disease (bone, ascites, pleural effusion) sites might have been overestimated.

Conversely, it should be emphasized that, 50% of patients were treated in third or more line; this, if confirmed by others, would suggest that antiangiogenic therapy may maintain a high activity even in heavily pretreated breast cancer.

Toxicity was acceptable with easily manageable side effects. We recorded one death due to cerebrovascular ischaemia, possibly related to treatment, in line with Miller *et al*, who report in their study a significant increase in cerebrovascular ischemia among patients receiving combined therapy (1.9% vs. 0.0%, *p *= 0.02) [8].

The main drawbacks of this study are the small number of patients and its retrospective nature, however, the trends observed in OS, as well as in PFS justify the need for further prospective randomized trials. Ongoing studies, such as the Ribbon and Ribbon 1, may confirm our results in the future.

The biweekly BEV/PAC combination seems to be active with acceptable toxicity and may represent an interesting option for salvage therapy in the "exhausted" patients with pretreated ABC. Furthermore this biweekly regimen has a clear advantage over the weekly one in terms of quality of life and resource sparing.

## Conclusion

The biweekly BEV/PAC combination seems to be active with acceptable toxicity and may represent an interesting option for salvage therapy-in this group of "exhausted" patients with pretreated ABC- that needs further investigation. Furthermore this biweekly regimen has a clear advantage over the weekly one in terms of quality of life and resource sparing.

## Competing interests

The authors declare that they have no competing interests.

## Authors' contributions

AA: Conception, Coordination, acquisition and interpretation of the data, manuscript preparation and approval of the final version. DD: Acquisition, analysis and interpretation of data, manuscript preparation and approval of the final version.

PK: Acquisition, analysis and interpretation of data, manuscript preparation and approval of the final version. SM: Acquisition, analysis and interpretation of data and approval of the final version. AK: Acquisition and analysis of data, approval of the final version. EK: Acquisition and analysis of data, approval of the final version.

DS: Acquisition and analysis of data, approval of the final version. MC: Acquisition and analysis of data, approval of the final version. GR: Coordination, preparation of the manuscript, approval of the final version.

All authors read and approved the final manuscript.

## Pre-publication history

The pre-publication history for this paper can be accessed here:

http://www.biomedcentral.com/1471-2407/9/338/prepub
